# Adjuvant therapy with Jianpi Huayu decoction improves overall and recurrence-free survival after hepatectomy for hepatocellular carcinoma: a retrospective propensity score-matching study

**DOI:** 10.3389/fphar.2023.1212116

**Published:** 2023-09-25

**Authors:** Rui Luo, Chongkai Fang, Chuyao Chen, Ying Zhang, Ruiwei Yao, Jinan Wang, Hanqian Shi, Kunliang Feng, Mingli Hu, Chong Zhong

**Affiliations:** ^1^ The First Clinical Medical School, Guangzhou University of Chinese Medicine, Guangzhou, China; ^2^ The First Affiliated Hospital, Guangzhou University of Chinese Medicine, Guangzhou, China; ^3^ Lingnan Medical Research Center of Guangzhou University of Chinese Medicine, Guangzhou, China; ^4^ Science and Technology Innovation Center, Guangzhou University of Chinese Medicine, Guangzhou, China; ^5^ Department of Surgery, Baiyun Hospital of the First Affiliated Hospital of Guangzhou University of Chinese Medicine, Guangzhou, China; ^6^ The Second Affiliated Hospital of Guizhou University of Chinese Medicine, Guizhou, China

**Keywords:** hepatocellular carcinoma, hepatectomy, tumor recurrence, traditional Chinese medicine, adjuvant therapy

## Abstract

Hepatocellular carcinoma (HCC) patients experience high rates of recurrence following hepatectomy. Many herbal preparations used in traditional Chinese medicine have been shown to improve the postoperative condition of cancer patients. This retrospective study examined the efficacy and safety of Jianpi Huayu decoction (JPHYD) as adjuvant therapy for HCC following hepatectomy. HCC patients received postoperative management according to Chinese Society of Clinical Oncology recommendations, either alone (Control group) or in addition to daily JPHYD (1 week in hospital and 3 months after release). To reduce selection bias, we performed 1:1 propensity score matching between the Control and JPHYD groups. The main endpoint was recurrence-free survival (RFS), and secondary endpoints included overall survival (OS) and adverse event frequency. A total of 207 patients meeting inclusion criteria were enrolled, 127 in the Control group and 80 in the JPHYD group. Patients were then propensity score-matched, yielding each group of 80. Recurrence-free survival rate was significantly higher in the JPHYD group than in the Control group at 1 year (67.9% vs. 38.1%), 2 years (39.1% vs. 26.2%), and 3 years (31.3% vs. 26.2%) following hepatectomy (HR 0.5666 [95%CI, 0.3655 to 0.8784]; *p* = 0.0066). Additionally, OS was significantly higher in the JPHYD group than the Control group at 1 year (94.3% vs. 81.9%), 2 years (76.4% vs. 58.8%), and 3 years (66.3% vs. 51.4%) following hepatectomy (HR 0.5199 [95%CI, 0.2849 to 0.9490]; *p* = 0.027). Adverse events frequencies did not differ between the two groups. In conclusion, JPHYD can safely improve RFS and OS following hepatectomy for HCC.

## 1 Introduction

Hepatocellular carcinoma (HCC) is the fifth most common cancer and the second leading cause of malignant death in Asia (Sung et al., 2021). In 2020, 72.5% of all reported cases worldwide were from Asia, with China alone accounting for 30% of newly diagnosed cases globally (Cao et al., 2021; Zhang et al., 2022). Due to the lack of donors, liver transplantation cannot be used as a routine treatment for early HCC in China, so hepatectomy is the preferred treatment (Zhang et al., 2021). However, the recurrence rate following hepatectomy is about 30%, resulting in reduced quality of life and shorter overall survival (OS) (Ercolani et al., 2003; Imamura et al., 2003). Therefore, it is critical to develop adjuvant therapies to reduce HCC recurrence and improve OS following hepatectomy (Heimbach et al., 2018).

Several clinicodemographic and presurgical factors have been identified that influence HCC recurrence risk after hepatectomy, such as tumor size, surgical method, and tumor stage, while other potential risk factors include hepatitis B/C virus (HBV/HCV) infection, liver cirrhosis, and microvascular invasion (MVI) (Zhang et al., 2018a; Liao et al., 2022). For high-risk patients, interventional therapy, targeted therapy, and (or) immunotherapy are used to reduce the recurrence rate (Zeng et al., 2022). In addition, several adjuvant therapies have proven broadly effective for temporarily reducing recurrence risk (Wang et al., 2018b; Zhang et al., 2018b; Chen et al., 2021a). However, the safest and most effective option for a given patient is still unclear, such as that with the lowest potential for liver dysfunction and drug resistance. Therefore, determining the most effective and safest adjuvant therapy or combination of adjuvant therapies for patients according to individual clinical and demographic characteristics is a major focus of current research on HCC treatment.

Traditional Chinese medicine (TCM) employs a variety of raw herbal mixtures or extracts to treat various ailments, and rigorous clinical studies have shown that some of these preparations can inhibit HCC growth, reduce the incidence of recurrence, and (or) improve the response to other treatments (either by mitigating toxic side effects or increasing dose sensitivity), thereby increasing survival time (Wei et al., 2022). Further, both *in vitro* and animal model studies have demonstrated that various TCM decoctions can prevent HCC cell proliferation, migration, and metastasis (Chen et al., 2018; Xie et al., 2022).

Jianpi Huayu decoction (JPHYD), a TCM formula based on *Jinkui Yaolue*, has been widely examined for the treatment of HCC. Our laboratory found that treatment of HCC cells with JPHYD blocked the signal pathways inducing epithelial–mesenchymal transition (EMT) (Chen et al., 2021b; Liu et al., 2022; Xie et al., 2022), a phenotypic transformation that enhances metastasis risk. We also demonstrated the effectiveness of JPHYD in prolonging the survival of HCC patients (Zhong et al., 2014). However, as is the case for other systemic, targeted, and immunotherapies, further study is required to identify those patients most likely to benefit from JPHYD.

Many studies on the clinical efficacy of TCMs for cancer and other diseases share two major limitations. First, diagnosis relies on the subjective reports of patients and the opinions of TCM physicians rather than the objective metrics provided by laboratory and imaging examinations. Second, TCM is frequently used for the prevention of cancer recurrence with other adjuvant treatments, so it is often not possible to establish a clear association between treatment and disease response. These limitations have to some extent hindered the promotion and use of TCM.

Therefore, in this study, we used propensity score-matching (PSM) to minimize patient selection bias and investigated the efficacy of JPHYD for improving RFS and OS rates among HCC patients following hepatectomy. In addition, we analyzed factors predictive of therapeutic response to identify patients or patient subgroups most likely to benefit from JPHYD.

## 2 Patients and methods

### 2.1 Study design and participants

Between March 2016 and March 2022, HCC patients receiving hepatectomy at the First Affiliated Hospital of Guangzhou University of Chinese Medicine (Guangzhou, China) were enrolled in this retrospective study. Patients receiving hepatectomy followed by conventional post-surgical management were considered candidates for the Control (non-JPHYD) group while those receiving additional JPHYD were considered candidates for the intervention group. In cases of tumor recurrence or high risk of recurrence after hepatectomy, anti-tumor treatment followed the Chinese Society of Clinical Oncology (CSCO) guidelines, including interventional therapy, targeted therapy, immunotherapy, or other systemic therapies. Some patients received two or more adjuvant therapies, referred to as comprehensive therapy.

The study was conducted in accordance with the Declaration of Helsinki and approved by the First Affiliated Hospital of Guangzhou University of Chinese Medicine Institutional Review Board and Ethics Committee (ethical approval number JY 2023-073). All post-hepatectomy treatments were based on CSCO Clinical Practice Guidelines.

### 2.2 Eligibility criteria

Inclusion criteria were as follows: (1) 18–75 years of age; (2) complete laboratory and imaging studies prior to surgery, and confirmed HCC diagnosis; (3) Child–Pugh class A/B live score; (4) no other antitumor treatments prior to surgery; (5) postoperative pathological diagnosis of HCC; (6) JPHYD for at least 3 months after hepatectomy in the intervention group; (7) no portal vein thrombus or extrahepatic metastasis observed before surgery in imaging examination.

The exclusion criteria were as follows: (1) recurrent HCC or other simultaneously occurring malignancies; (2) serious major organ dysfunction; (3) receiving other main components of TCM treatment; (4) missing clinical and demographic data; (5) death by other causes.

### 2.3 Radical hepatectomy

Hepatectomy was conducted as previously described (Zhong et al., 2014; Fang et al., 2023). The resection margins met R0 resection criteria.

### 2.4 JPHYD treatment

Patients in the intervention group received daily JPHYD for at least 1 week in hospital and 3 months after release. The components of JPHYD include *Salvia miltiorrhiza* Bge. (Danshen), *Atractylodes macrocephala* Koidz. (Baizhu), *Bupleurum chinense* DC. (Chaihu), *Paeonia suffruticosa* Andr. (Mudanpi), *Dioscorea oppositifolia* L. (Shanyao), *Curcuma phaeocaulis* Valeton (Ezhu), *Poria cocos* (Schw.) Wolf (Fuling), *Panax ginseng* C.A.Mey. (Renshen), *Curcuma longa* L. (Yujin), *Glycyrrhiza uralensis* Fisch. ex DC. (Gancao) (Table 1). All components can be found in *The Chinese Pharmacopoeia*.

**TABLE 1:** Components of Jianpi Huayu decoction.

Latin name	Herb name in Chinese	Scientific name	Family	Parts used	Weight (g)
*Salviae Miltiorrhizae Radix Et Rhizoma*	Danshen	*Salvia miltiorrhiza* Bge.	Lamiaceae	dry root and rhizome	15
*Atractylodis Macrocephalae Rhizoma*	Baizhu	*Atractylodes macrocephala* Koidz.	Asteraceae	dry rhizome	15
*Bupleuri Radix*	Chaihu	*Bupleurum chinense* DC.	Apiaceae	dry root	15
*Moutan Cortex*	Mudanpi	*Paeonia suffruticosa* Andr.	Paeoniaceae	dry root bark	10
*Dioscoreae Rhizoma*	Shanyao	*Dioscorea oppositifolia* L.	Dioscoreaceae	dry rhizome	12
*Curcumae Rhizoma*	Ezhu	*Curcuma phaeocaulis* Valeton	Zingiberaceae	dry root tuber	10
*Poria*	Fuling	*Poria cocos* (Schw.) Wolf	Polyporaceae	sclerotia	15
*Ginseng Radix Et Rhizoma*	Renshen	*Panax ginseng* C.A.Mey.	Araliaceae	dry root and rhizome	20
*Curcumae Radix*	Yujin	*Curcuma longa* L.	Zingiberaceae	dry root tuber	10
*Glycyrrhizae Radix Et Rhizoma*	Gancao	*Glycyrrhiza uralensis* Fisch. ex DC.	Fabaceae	dry root and rhizome	6

The herbs were purchased from the First Affiliated Hospital of Guangzhou University of Chinese Medicine (Guangzhou, China). The herbs were identified and stored by experts from the College of Pharmacy, the First Affiliated Hospital of Guangzhou University of Chinese Medicine (Guangzhou, China). As clinical patients may exhibit other symptoms unrelated to liver cancer (such as throat itchiness, headache, and reduced urination), our experienced TCM practitioners added additional low-dose herbal components to the herbal prescription. These supplementary low-dose herbal components are unrelated to the patient’s liver cancer condition, and the primary ingredient remains JPHYD. In the Supplementary Material, we have provided explanations on which specific herbal components should be added based on the patient’s individual symptoms.

### 2.5 Quality control of JPHYD

Patients who purchase JPHYD will be informed about the decoction process. The method involves soaking the herbs for 40 min, adding 10 times the amount of water, boiling for 40 min, and performing two rounds of boiling. This process results in a final volume of 150 ml of liquid. The same decoction process is employed to enhance the quality control elements of this study. After LC-MS/MS analysis, the active metabolites of JPHYD were identified as *Poricoic* acid A, *Ginsenoside* Rg_1_, *Tanshinone* ⅡA, and *Saikosaponin* A (the detailed detection method can be found in the Supplementary Material).

### 2.6 Data collection

Baseline demographic and clinical characteristics, including sex, age, Barcelona Clinic Liver Cancer (BCLC) stage, number of tumor(s), tumor distribution, maximum tumor diameter, tumor differentiation state, MVI, cirrhosis, HBV/HCV infection, HBV DNA, indocynine green rate 15 (ICG 15), Child–Pugh stage, and serum biomarkers, were gathered by two researchers with double checking. To reduce possible selection bias and potential confounders, we performed 1:1 matching between Control and JPHYD groups using the PSM application of R (version 4.0.5: R Foundation, Vienna, Austria). The PSM application uses a logistic regression model to match all (22) baseline characteristics by the optimization method.

### 2.7 Endpoints

RFS, defined as the time from hepatectomy to observed recurrence, was the primary endpoint, while OS, defined as the time from hepatectomy to death, was the secondary endpoint. For patients remaining recurrence-free at final follow-up, we censored patient states as event-free and alive for the analysis. All adverse events (AEs) were recorded after hepatectomy and graded according to the National Cancer Institute Common Terminology Criteria for Adverse Events version 4.03.

### 2.8 Statistical analysis

Categorical clinicodemographic variables are expressed as number of cases and percentages which was performed by the chi-square test, while continuous clinicodemographic variables are expressed as the median and interquartile range (IQR) which was performed by the student’s *t*-test. The RFS and OS rates were calculated by Kaplan-Meier survival analysis and compared between groups by log-rank test. Cox proportional hazard models were constructed to calculate hazard ratios (HRs) with 95% confidence intervals (CIs). Survival curves were drawn and analyzed using R. The locations of tumor recurrence and forest plot were drawn and analyzed using GraphPad Prism version 8.0.1 for Windows (GraphPad Software San Diego, CA, USA). Subgroup analyses included sex, age, BCLC stage, number of tumor(s), tumor distribution, maximum tumor diameter, tumor differentiation state, MVI, cirrhosis, AFP, HBV infection, HBV DNA, ICG 15, Child–Pugh stage, and comprehensive therapy as potential prognostic factors. These analyses were conducted using SPSS software version 25 (Chicago, IL, USA). A *p* < 0.05 was considered statistically significant for all tests.

## 3 Results

### 3.1 Patient characteristics and treatment administration

**FIGURE 1:**
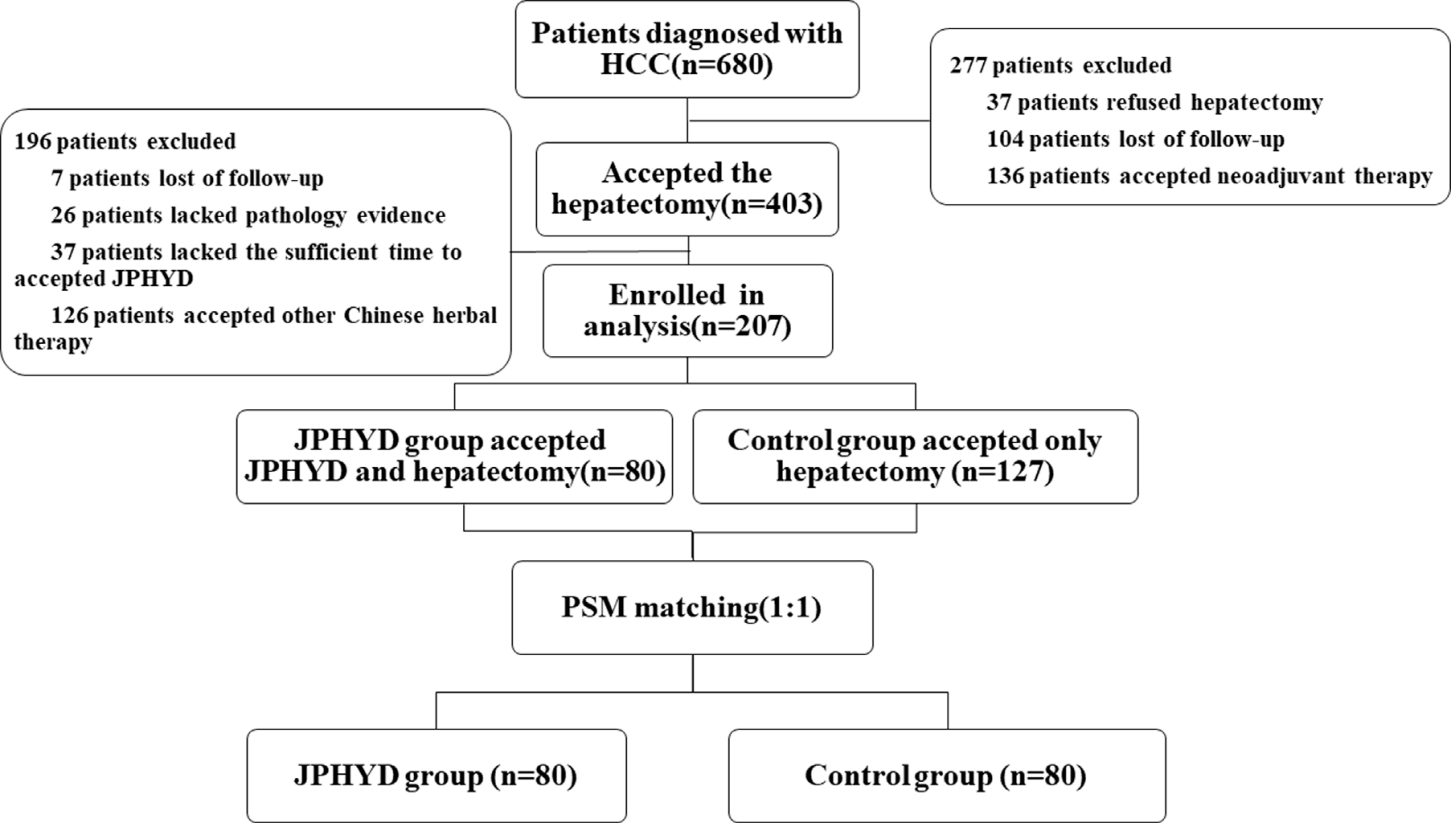
Flowchart ofshows the patients’ selection process. HCC, Hepatocellular Carcinoma; JPHYD, Jianpi Huayu Decoction; PSM, Propensity Score-matching.

**FIGURE 2:**
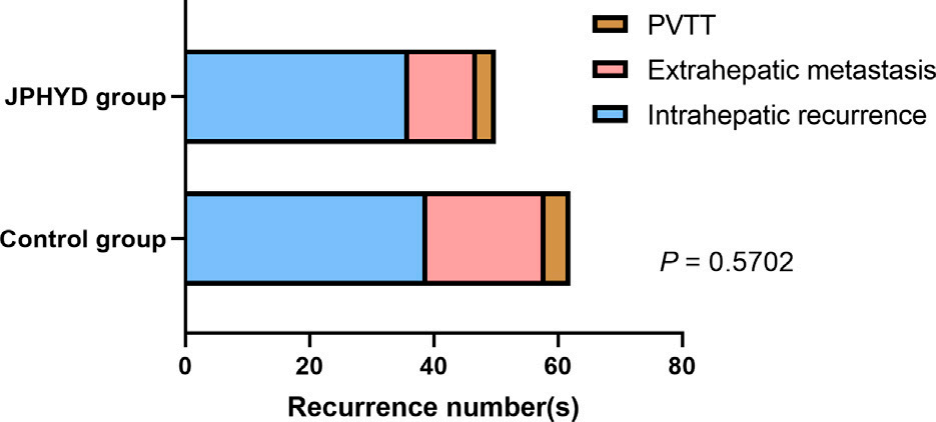
Tumor recurrence location(s) in non-JPHYD (Control) and JPHYD groups. PVTT, portal vein tumor thrombus.

From March 2016 to March 2022, a total of 680 patients were diagnosed with HCC at the First Affiliated Hospital of Guangzhou University of Chinese Medicine (Guangzhou, China), of which 403 received hepatectomy. Of these hepatectomy patients, 196 who received other Chinese herbal treatments or other reasons were excluded, while the remaining 207 patients met our inclusion criteria (Figure 1). This cohort included 127 patients receiving conventional post-surgical management as recommended by CSCO (Control group) and 80 patients receiving JPHYD in addition to CSCO-recommended treatments. However, the clinicodemographic characteristics had several significant differences in subgroups to influence the balance between the two groups (Table 2). Therefore, Control group patients were then propensity score-matched to the 80 JPHYD group patients yielding each group of 80 well matched for clinicodemographic characteristics (Table 3). There were no significant differences in most measured parameters (all *p* > 0.05), except for serum total bilirubin (TBil). However, in both groups, the median TBil was below 34.2 μmol/L, the upper limit of the normal range. Some patients received comprehensive therapy (Table 4) including chemical intervention therapy (CIT) which means transarterial chemoembolization (TACE) combined with hepatic arterial infusion chemotherapy (HAIC), targeted-therapy plus immune therapy (TPI), or CIT combined with TPI therapy and radio frequency ablation (RFA). However, there was no significant difference in the proportion receiving each comprehensive treatment between the Control and JPHYD groups. In the Control group, there were 39 cases of intrahepatic recurrence, 19 of extrahepatic metastasis, and 4 of portal vein tumor thrombus. In the JPHYD group, there were 36 cases of intrahepatic recurrence, 11 of extrahepatic metastasis, and 3 cases of portal vein tumor thrombus (Figure 2).

**TABLE 2:** Baseline characteristics of patients before PSM.

Characteristics		Overall (n = 207)	Control group (n = 127)	JPHYD group (n = 80)	*P*
Gender	Male	189 (91.3%)	119 (93.7%)	70 (87.5%)	0.198
	Female	18 (8.7%)	8 (6.3%)	10 (12.5%)	
Age	<60	131 (63.3%)	81 (63.8%)	50 (62.5%)	0.97
	≥60	76 (36.7%)	46 (36.2%)	30 (37.5%)	
BCLC	A	131 (63.3%)	81 (63.8%)	50 (62.5%)	0.97
	B	76 (36.7%)	46 (36.2%)	30 (37.5%)	
Number of tumor(s)	Single	142 (68.6%)	85 (66.9%)	57 (71.2%)	0.618
	Multiple	65 (31.4%)	42 (33.1%)	23 (28.7%)	
Tumor Distribution	One-side	182 (87.9%)	107 (84.3%)	75 (93.8%)	0.068
	Two-side	25 (12.1%)	20 (15.7%)	5 (6.2%)	
Maximum diameter of tumor (cm)	≤8 cm	131 (63.3%)	73 (57.5%)	58 (72.5%)	0.042
	>8 cm	76 (36.7%)	54 (42.5%)	22 (27.5%)	
Differentiation of tumor	High	28 (13.5%)	9 (7.1%)	19 (23.8%)	0.002
	Middle	101 (48.8%)	64 (50.4%)	37 (46.2%)	
	Low	78 (37.7%)	54 (42.5%)	24 (30.0%)	
MVI	No	132 (63.8%)	76 (59.8%)	56 (70.0%)	0.183
	Yes	75 (36.2%)	51 (40.2%)	24 (30.0%)	
Cirrhosis	No	90 (43.5%)	61 (48.0%)	29 (36.2%)	0.128
	Yes	117 (56.5%)	66 (52.0%)	51 (63.7%)	
Hepatitis B infection	No	28 (13.5%)	20 (15.7%)	8 (10.0%)	0.333
	Yes	179 (86.5%)	107 (84.3%)	72 (90.0%)	
Hepatitis C infection	No	204 (98.6%)	125 (98.4%)	79 (98.8%)	1
	Yes	3 (1.4%)	2 (1.6%)	1 (1.2%)	
HBV DNA	≤1,000	116 (56.0%)	61 (48.0%)	55 (68.8%)	0.005
	>1,000	91 (44.0%)	66 (52.0%)	25 (31.2%)	
ICG 15	≤10%	183 (88.4%)	116 (91.3%)	67 (83.8%)	0.151
	>10%	24 (11.6%)	11 (8.7%)	13 (16.2%)	
AFP	<400	138 (66.7%)	79 (62.2%)	59 (73.8%)	0.118
	≥400	69 (33.3%)	48 (37.8%)	21 (26.2%)	
Child-Pugh stage	A	201 (97.1%)	123 (96.9%)	78 (97.5%)	1
	B	6 (2.9%)	4 (3.1%)	2 (2.5%)	
Serum biomarker	Tbil	11.10 [8.20, 14.50]	12.00 [8.95, 15.10]	9.45 [6.88, 13.03]	0.002
	WBC	6.35 [5.14, 7.59]	6.48 [5.18, 7.75]	6.15 [4.89, 7.18]	0.267
	NEU	3.79 [2.90, 4.86]	3.87 [2.89, 4.93]	3.58 [2.92, 4.72]	0.413
	HGB	144.00 [131.00, 153.00]	146.00 [132.90, 157.00]	137.00 [126.50, 148.50]	0.002
	PLT	197.00 [149.50, 250.50]	204.00 [155.50, 259.50]	186.50 [136.75, 236.75]	0.056
	ALT	33.40 [22.00, 51.50]	36.00 [24.80, 57.45]	30.00 [19.75, 44.25]	0.022
	ALB	41.70 [39.00, 44.15]	42.30 [39.80, 44.45]	41.45 [37.88, 43.02]	0.019
	PT	11.70 [11.20, 12.50]	11.70 [11.20, 12.50]	11.65 [11.10, 12.53]	0.971
	CRE	76.70 [68.10, 87.00]	76.50 [68.20, 85.95]	78.50 [68.00, 90.25]	0.394

Abbreviations: ICG 15, indocynine green rate 15; AFP, alpha-fetoprotein; TBil, total bilirubin; WBC, white blood cells; NEU, neutrophil; HGB, hemoglobin; PLT, platelet count; ALT, alanine aminotransferase; ALB, albumin; PT, prothrombin time; CRE, creatinine.

**TABLE 3:** Baseline characteristics of patients after PSM.

Characteristics		Overall (n = 160)	Control group (n = 80)	JPHYD group (n = 80)	*P*
Gender	Male	142 (88.8%)	72 (90.0%)	70 (87.5%)	0.802
	Female	18 (11.2%)	8 (10.0%)	10 (12.5%)	
Age	<60	96 (60.0%)	46 (57.5%)	50 (62.5%)	0.628
	≥60	64 (40.0%)	34 (42.5%)	30 (37.5%)	
BCLC	A	102 (63.7%)	52 (65.0%)	50 (62.5%)	0.869
	B	58 (36.2%)	28 (35.0%)	30 (37.5%)	
Number of tumor(s)	Single	111 (69.4%)	54 (67.5%)	57 (71.2%)	0.732
	Multiple	49 (30.6%)	26 (32.5%)	23 (28.7%)	
Tumer Distribution	One-side	149 (93.1%)	74 (92.5%)	75 (93.8%)	1
	Two-side	11 (6.9%)	6 (7.5%)	5 (6.2%)	
Maximum diameter of tumor (cm)	≤8 cm	111 (69.4%)	53 (66.2%)	58 (72.5%)	0.493
	>8 cm	49 (30.6%)	27 (33.8%)	22 (27.5%)	
Differentiation of tumor	High	27 (16.9%)	8 (10.0%)	19 (23.8%)	0.067
	Middle	80 (50.0%)	43 (53.8%)	37 (46.2%)	
	Low	53 (33.1%)	29 (36.2%)	24 (30.0%)	
MVI	No	108 (67.5%)	52 (65.0%)	56 (70.0%)	0.613
	Yes	52 (32.5%)	28 (35.0%)	24 (30.0%)	
Cirrhosis	No	63 (39.4%)	34 (42.5%)	29 (36.2%)	0.517
	Yes	97 (60.6%)	46 (57.5%)	51 (63.7%)	
Hepatitis B infection	No	19 (11.9%)	11 (13.8%)	8 (10.0%)	0.625
	Yes	141 (88.1%)	69 (86.2%)	72 (90.0%)	
Hepatitis C infection	No	158 (98.8%)	79 (98.8%)	79 (98.8%)	1
	Yes	2 (1.2%)	1 (1.2%)	1 (1.2%)	
HBV DNA	≤1,000	100 (62.5%)	45 (56.2%)	55 (68.8%)	0.142
	>1,000	60 (37.5%)	35 (43.8%)	25 (31.2%)	
ICG 15	≤10%	138 (86.2%)	71 (88.8%)	67 (83.8%)	0.491
	>10%	22 (13.8%)	9 (11.2%)	13 (16.2%)	
AFP	<400	115 (71.9%)	56 (70.0%)	59 (73.8%)	0.725
	≥400	45 (28.1%)	24 (30.0%)	21 (26.2%)	
Child-Pugh stage	A	158 (98.8%)	80 (100.0%)	78 (97.5%)	0.477
	B	2 (1.2%)	0 (0.0%)	2 (2.5%)	
Serum biomarker	Tbil	10.60 [7.77, 13.60]	11.90 [8.47, 14.08]	9.45 [6.88, 13.03]	0.038
	WBC	6.35 [4.93, 7.41]	6.48 [4.96, 7.54]	6.15 [4.89, 7.18]	0.51
	NEU	3.70 [2.81, 4.72]	3.79 [2.73, 4.65]	3.58 [2.92, 4.72]	0.878
	HGB	139.50 [127.75, 150.00]	143.50 [128.75, 152.00]	137.00 [126.50, 148.50]	0.174
	PLT	189.50 [147.00, 244.00]	200.00 [151.50, 249.50]	186.50 [136.75, 236.75]	0.281
	ALT	31.50 [20.00, 47.72]	32.50 [21.50, 51.40]	30.00 [19.75, 44.25]	0.318
	ALB	41.55 [38.68, 43.82]	41.60 [39.50, 44.30]	41.45 [37.88, 43.02]	0.154
	PT	11.70 [11.10, 12.60]	11.70 [11.07, 12.60]	11.65 [11.10, 12.53]	0.815
	CRE	76.30 [68.00, 87.00]	76.00 [67.73, 84.85]	78.50 [68.00, 90.25]	0.243

**TABLE 4:** Comprehensive adjuvant treatments after hepatectomy in the Control group and JPHYD group.

	JPHYD group	Control group	*P*
CIT*	6	0	0.016
TPI*	6	0	
CIT combined TPI*	16	6	
CIT combined RFA*	1	3	
CIT combined TPI and RFA*	0	1	

CIT, chemical intervention therapy; TPI, targeted-therapy plus immune therapy; RFA, radio frequency ablation; *, no statistical difference within the Control group and JPHYD group.

### 3.2 Efficacy analysis

**FIGURE 3:**
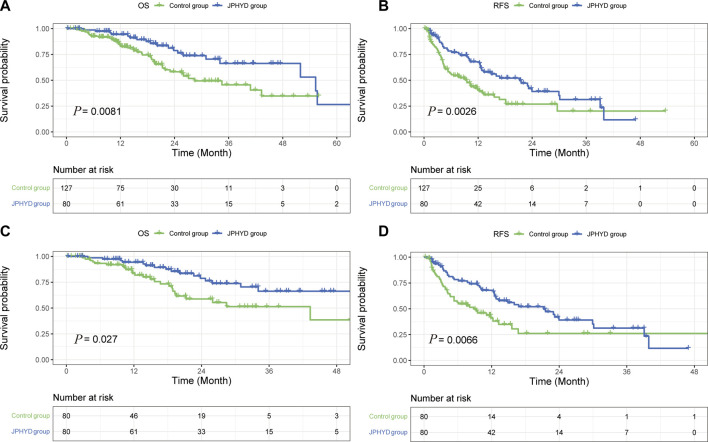
Kaplan–Meier survival curves of post-hepatectomy patients in Control and JPHYD groups. **(A, B)** Overall survival (OS) **(A)** and recurrence-free survival (RFS) **(B)** prior to propensity score-matching (PSM). **(C, D)** OS **(C)** and RFS **(D)** after PSM.

At the final follow-up, there were 19 patient deaths and 41 recurrences in the JPHYD group and 42 patient deaths and 71 recurrences in the entire (unmatched) Control group. Overall survival rates were significantly higher in the JPHYD group than the entire Control group at 1 year (94.3% vs. 83.6%), 2 years (76.4% vs. 58.2%), and 3 years (66.3% vs. 45.6%) post-hepatectomy (HR 0.4933 [95%CI, 0.2985 to 0.8153]; *p* = 0.0081) (Figure 3A). Moreover, the median OS was significantly longer in the JPHYD group than in the entire Control group (55.3 months vs. 28.43 months). Additionally, RFS was significantly higher in the JPHYD group than the entire Control group at 1 year (67.9% vs. 41%), 2 years (39.1% vs. 26.9%), and 3 years (31.3% vs. 20.2%) post-hepatectomy (HR 0.5662 [95%CI, 0.3909 to 0.8201]; *p* = 0.0026) (Figure 3B), and median RFS was longer in the JPHYD group than the unmatched Control group (21.23 months vs. 9.267 months).

After PSM, there were 19 patient deaths and 41 recurrences in the JPHYD group compared to 25 patient deaths and 44 recurrences in the matched Control group. OS was still significantly higher in the JPHYD group compared to the Control group at 1 year (94.3% vs. 81.9%), 2 years (76.4% vs. 58.8%), and 3 years (66.3% vs. 51.4%) post-hepatectomy (HR 0.5199 [95%CI, 0.2849 to 0.9490]; *p* = 0.027) (Figure 3C), and median OS was longer in JPHYD group than the matched Control group (55.3 months vs. 43.3 months). Recurrence-free survival rate was also significantly higher in the JPHYD group compared to the matched Control group at 1 year (67.9% vs. 38.1%), 2 years (39.1% vs. 26.2%), and 3 years (31.3% vs. 26.2%) post-hepatectomy (HR 0.5666 [95%CI, 0.3655 to 0.8784]; *p* = 0.0066) (Figure 3D), and median RFS was longer in the JPHYD group than the matched Control group (21.23 months vs. 8.83 months). Thus, adjunct JPHYD treatment following hepatectomy demonstrated significant OS and RFS advantages, even after PSM.

**FIGURE 4:**
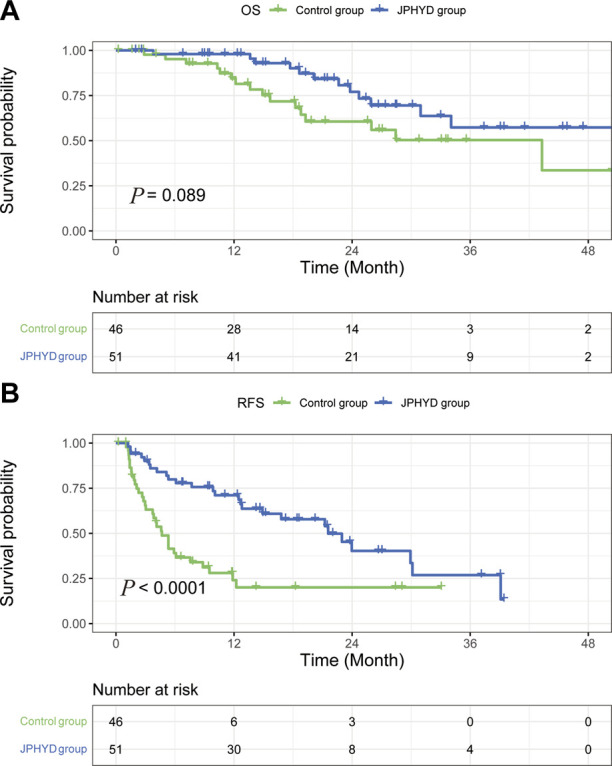
Kaplan–Meier survival curves of cirrhosis patients in Control and JPHYD groups after PSM. **(A)** Overall survival (OS). **(B)** Recurrence-free survival (RFS).

**FIGURE 5:**
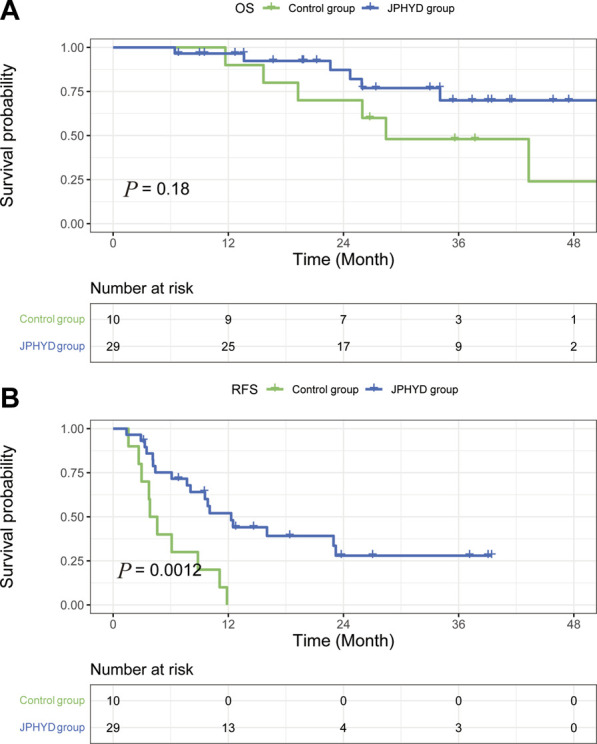
Kaplan–Meier survival curves of post-hepatectomy patients who accepted comprehensive therapy in Control and JPHYD groups after PSM. **(A)** Overall survival (OS). **(B)** Recurrence-free survival (RFS).

Among patients with cirrhosis, OS rate did not differ significantly between JPHYD and Control groups at 1 year (97.9% vs. 81.4%), 2 years (77.0% vs. 60.6%), and 3 years (57.3% vs. 50.3%) post-hepatectomy (HR 0.5366 [95%CI, 0.2551 to 1.129]; *p* = 0.089) (Figure 4A). However, the RFS rate was still significantly higher in the JPHYD group compared to the Control group at 1 year (71.1% vs. 24%), 2 years (40.3% vs. 20%), and 3 years (26.8% vs. 20%) post-hepatectomy (HR 0.3806 [95%CI, 0.2191 to 0.6610]; *p* < 0.0001) (Figure 4B). Among patients receiving comprehensive therapy as well, OS did not differ significantly between JPHYD and Control groups at 1 year (96.5% vs. 90%), 2 years (82% vs. 70%), and 3 years (69.9% vs. 48%) post-hepatectomy (HR 0.4810 [95%CI, 0.1455 to 1.590]; *p* = 0.178) (Figure 5A). Also, the RFS rate was higher in the JPHYD group than the Control group at 6 months (71.6% vs. 30%) and 1 year (48.1% vs. 0%) post-hepatectomy (HR 0.3094 [95%CI, 0.1086 to 0.8814]; *p* = 0.0012) (Figure 5B). Thus, adjunct JPHYD treatment following hepatectomy demonstrated a significant RFS advantage even among patients with cirrhosis and those receiving comprehensive treatment(s).

**FIGURE 6:**
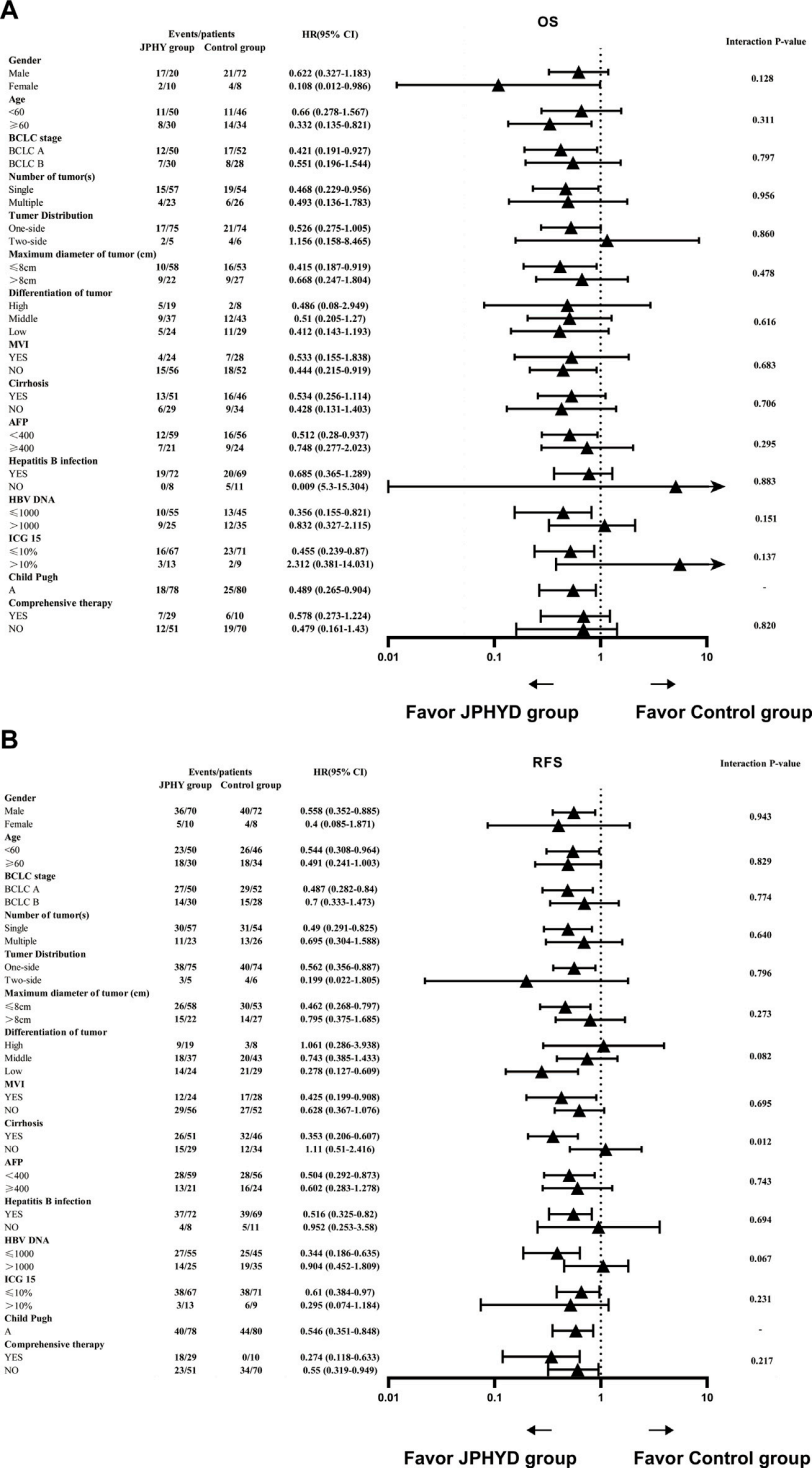
Hazard ratios (HRs) for OS and RFS according to baseline prognostic factors. **(A)** OS. **(B)** RFS. Each triangle represents the estimated HR, the horizontal lines represent the 95% CIs. A two-tailed *p* < 0.05 was considered statistically significant.

Results of further subgroup analyses using the Cox proportional hazards model are shown in Figure 6. Treatment with JPHYD yielded significant OS advantages for females, patients ≥ 60 years, and patients with BCLC A, single tumor, maximum tumor diameter ≤ 8 cm, without MVI, AFP < 400, HBV DNA ≤ 1,000, ICG ≤ 10%, or Child-Pugh A grade disease (Figure 6A). Further, JPHYD yielded significant RFS advantages for males, patients aged < 60 years, patients with BCLC A, single tumor, tumor distribution on one-side, maximum tumor diameter ≤ 8 cm, low-grade tumor differentiation, presence of MVI and cirrhosis, AFP < 400, HBV infection, HBV DNA ≤ 1,000, ICG ≤ 10%, or Child-Pugh A grade disease, regardless of whether they received comprehensive therapy (Figure 6B). Although the interaction *p*-value was not statistically significant, the RFS subgroup analysis also showed a difference in the group with cirrhosis.

### 3.3 Safety analysis

Adverse events frequencies did not differ significantly between Control and JPHYD groups (Table 5), and the majority of AEs in both groups were grade 1 to 2. There were also no reports of death related to JPHYD or hepatectomy.

**TABLE 5:** Summary of treatment-related adverse events.

	JPHYD group (n = 80)			Control group (n = 80)			*p* Value
	Grade 1	Grade 2	Grade 3 and grade 4	Grade 1	Grade 2	Grade 3 and grade 4	
Hypoproteinemia	22	7	0	20	9	0	0.557
Hepatocyte dysfunction	23	14	1	28	5	4	0.051
Coagulation disorders	2	0	0	4	2	0	0.346
Jaundice	5	1	0	17	18	7	0.134
Diarrhoea	6	0	0	12	1	0	0.485
Emesis	19	0	0	4	0	0	-
Infection	8	2	0	3	2	1	0.298
Hypertension	0	0	0	3	0	0	-
Cough	1	1	0	4	1	0	0.427
Anemia	17	4	4	36	11	1	0.076
Edema	1	0	0	9	1	0	0.740
Pain	21	2	0	16	1	0	0.738
Insomnia	1	0	0	4	1	0	0.624
Creatinine elevation	0	0	0	0	2	0	-
Haemorrhage	0	1	0	2	0	3	0.05

## 4 Discussion

Radical surgery and liver transplantation are undoubtedly the best treatments for early-stage HCC. However, due to the shortage of liver donors, most patients in China receive liver resection treatment. Therefore, reducing the recurrence rate and improving survival after hepatectomy is currently the fundamental challenge in hepatic cancer treatment. Postoperative adjuvant therapy such as interventional therapy, targeted therapy, and immunotherapy is beneficial to inhibit tumor recurrence following hepatectomy, but the optimal combination plan for specific populations is still being explored (Tung-Ping Poon et al., 2000). Several adjuvant therapies have proven broadly beneficial for prolonging survival, but many patients cannot tolerate the associated adverse reactions, so the selection must be considered carefully (Lencioni et al., 2016). In clinical practice, first-line and adjuvant treatment methods are coordinated through multidisciplinary cooperation to achieve comprehensive therapy. TCM is based on natural products with documented efficacy and safety, so could be an important part of the comprehensive anti-tumor therapy for HCC. Here we demonstrate that JPHYD as postoperative adjuvant therapy can significantly prolong recurrence-free and overall survival following hepatectomy for HCC.

Our research group has focused on JPHYD based on clinical trials and laboratory studies showing antiproliferative, antitumor, and antimetastatic efficacy against HCC (Zhong et al., 2014). The main bioactivities of the JPHYD include “fortifying the spleen”, “invigorating blood”, and “dissolving stasis”. The basic components of the formula, including *Atractylodes macrocephala* Koidz., *Panax ginseng* C.A.Mey., *Dioscorea oppositifolia* L., and *Poria cocos* (Schw.) Wolf, could relieve anemia, improve appetite, and alleviate fatigue by “fortifying the spleen” and “replenishing qi” (Tian et al., 2016; Chen et al., 2019; Ma et al., 2021). According to TCM theory, “blood stasis” or “phlegm rheum” is a core pathomechanism of HCC or “ji”. Therefore, TCMs that promote “blood circulation” are used clinically for HCC, such as *Paeonia suffruticosa* Andr., *Salvia miltiorrhiza* Bge., *Curcuma longa* L., and *Curcuma phaeocaulis* Valeton (Wang et al., 2014; Wu et al., 2022). Additionally, JPHYD incorporates *Bupleurum chinense* DC. which is a “Shaoyang meridian” drug that can “soothe the liver and relieve urgency”, thereby alleviating postoperative pain and perioperative symptoms (Xu et al., 2016). *Glycyrrhiza uralensis* Fisch. ex DC., the most widely used Chinese medicine in clinical practice, can reduce the pharmacological toxicity of drugs, thereby enhancing treatment safety (Li et al., 2019). Despite these promising results, further clinical studies are required to identify the optimal treatment protocol and most suitable target patient population. Therefore, this study focuses on the efficiency of JPHYD in clinical and provides a foundation for future clinical trials and studies on the indications for the fixed ingredients.

A meta-analysis concluded that there was insufficient evidence for the anticancer efficacy of TCM due to poor methodological quality (Ma et al., 2017). Therefore, PSM was used in this study to reduce bias caused by differences in patient characteristics, thereby strengthening the statistical power of this relatively small cohort (80 per group). In addition, although the methodological and analytic standards of TCM research are constantly improving, TCM is still a highly subjective field and thus greatly influenced by the proficiency of doctors; moreover, there is no objective evaluation standard for patients (Wang et al., 2016). Hence, we adopted inclusion criteria consistent with mainstream prospective studies to ensure scientific rigor.

The efficacy of JPHYD was significant both when compared to a heterogenous Control group and a matched Control group selected by PSM. The therapeutic effect may stem from inhibition of tumor cell proliferation and metastases. In addition, the JPHYD may enhance the efficacy of other adjuvant treatments or promote compliance by reducing side effects. Adjuvant use of Chinese medicines requires patients to return to the hospital for reexamination once every 1–2 weeks, which may maintain overall treatment compliance. Collectively, these effects may have contributed to prolonged RFS and OS following hepatectomy.

Consistent with previous studies, HCC recurrence occurred mainly within the liver with multiple satellite lesions. Thus, preventing intrahepatic metastasis is crucial for reducing postoperative recurrence (Zhai et al., 2018; Llovet et al., 2021). While JPHYD did not significantly reduce recurrence numbers at final follow-up or suppress extrahepatic metastasis, it significantly prolonged the time to recurrence, which in turn could improve the survival time of HCC patients. Therefore, JPHYD may be even more beneficial when combined with other adjuvant therapies that can inhibit metastasis, especially for patients at high risk of recurrence. In this study, JPHYD was in fact combined with various adjuvant therapies such as TACE, HAIC, targeted therapy, or immunotherapy. As stated, it is possible that JPHYD enhanced the efficacies of these treatments or exerted detoxifying effects. Indeed, the combination of Chinese medicines and TACE has been shown to prolong the survival time of patients with unresectable HCC, and preclinical experiments indicate that some TCM preparations can reshape the immune microenvironment of HCC, improve the therapeutic effect of low-dose sorafenib, and increase liver cancer cell sensitivity to sorafenib (Tang et al., 2016; Yang et al., 2020). In addition, TACE combined with multimodal immunotherapy resulted in longer OS than single-agent immune checkpoint inhibitor (ICI) treatment, while TACE, sorafenib, and ICIs demonstrated greater efficacy when combined than when administered separately (De Toni, 2020; Kelley et al., 2021). In accord with these findings, JPHYD reduced the early RFS rate among patients receiving comprehensive therapy and also increased OS over 3 years, although this latter effect did not reach statistical significance.

Subgroup analysis also revealed that JPHYD can prolong the RFS of patients with liver cirrhosis. This could be attributed to the anti-fibrotic properties of bioactive TCM metabolites such as flavonoids, saponins, polysaccharides, and alkaloids, which inhibit liver inflammation and regulate the synthesis and secretion of pro-fibrotic factors (Shan et al., 2019). Studies have also shown that entecavir combined with TCM can reverse liver fibrosis (Wang et al., 2018a; Cheng et al., 2022), and improving cirrhosis can both reduce tumor recurrence rate and prolong survival time (Chen et al., 2016). According to TCM theory, liver cirrhosis caused by portal hypertension is characterized as “blood stasis”, which is a strong risk factor for HCC, while the herbs *Salvia miltiorrhiza* Bge., *Curcuma phaeocaulis* Valeton, and *Curcuma longa* L. in JPHYD can promote blood circulation, activate blood, and resolve stasis (Marasco et al., 2019). Further, Tanshinol in *Salvia miltiorrhiza* Bge. can promote local blood circulation in the liver and improve portal hypertension caused by liver cirrhosis (Tian and Xu, 2009; Joe et al., 2012; Zhao et al., 2012). Therefore, JPHYD may prolong RFS by improving liver cirrhosis.

This study confirms that JPHYD is a safe and tolerable adjuvant treatment for postoperative HCC patients as AE frequency did not differ from Controls during the 3-month administration period. Thus, the AEs observed were likely caused by liver resection surgery. Also, the majority of AEs during hospitalization were mild and manageable, and no serious AEs were reported after discharge among patients taking JPHYD. In fact, some TCMs can improve patient compliance and increase the efficacy of adjuvant therapy. Further study is required to assess these potential benefits of JPHYD.

This study has several limitations. First, the retrospective, single-center study design and relatively small sample size could introduce selection bias and reduce the statistical power of some comparisons. Further studies should therefore examine the effectiveness of JPHYD as a fixed proprietary Chinese medicine with multicenter-clinical trial. Second, although PSM was used to balance the basic characteristics of patients, baseline the TBil group still differed between groups, potentially confounding the final results. Third, patients with high-risk factors received other adjuvant therapies, again confounding statistical outcomes. Fourth, subgroup analysis of patients for OS with comprehensive treatment and liver cirrhosis did not reveal a statistically significant group difference, possibly due to insufficient use of TCM. Future research should examine the dose–response relationship of JPHYD for HCC patients following hepatectomy. Finally, given the retrospective study, the herbal medicine decoctions had already been consumed by the patients during the design of this study, making it unfeasible for us to monitor the fingerprint profiles of the specific batches used in their treatment. Although we have previously validated the metabolites of some herbal medicines in our experiments, we acknowledge that our efforts may not fully meet the requirements outlined in the ConPhyMP statement for type A extracts. In our future studies, we are committed to enhancing our efforts to characterize the profiles of herbal decoctions used in our clinical investigations, aligning with the significance emphasized in the ConPhyMP statement, for a more comprehensive representation of our research findings.

JPHYD improved the RFS and OS of HCC patients following hepatectomy, possibly by improving liver cirrhosis and enhancing the efficacies of other adjuvant therapies. Larger randomized controlled trials are necessary to confirm the survival benefits of JPHYD in specific patient subgroups.

## Data Availability

The raw data supporting the conclusion of this article will be made available by the authors, without undue reservation.
