# Supplementary material for: Adjuvant therapy with Jianpi Huayu decoction improves overall and recurrence-free survival after hepatectomy for hepatocellular carcinoma: a retrospective propensity score-matching study
Source: Front Pharmacol. 2023 Sep 25;14:1212116. doi: 10.3389/fphar.2023.1212116 (PMC10561391; doi:10.3389/fphar.2023.1212116)
Supplement: Supplementary file 1 [file DataSheet1.docx]

# LC-MS/MS analysis of Jianpi Huayu Decoction (JPHYD)

## Reagents and Drugs

The reference metabolites, *Poricoic* acid A (no. AF20082214), *Ginsenoside* Rg1 (no. AF20030231), *Tanshinone* ⅡA (no. AF9040812), *Saikosaponin* A (no. AF20062251), were purchased from Chengdu Alfa Biotechnology Co., Ltd (Chengdu, China) with a purity exceeding 98%. Merck (Darmstadt, Germany) supplied acetonitrile (HPLC grade), Kermel Chemical Reagent Co., Ltd (Tianjin, China) provided formic acid (HPLC grade), and A.S. Watson Group (Hong Kong, China) furnished ultra-pure water. Additionally, all herbal materials were supplied by Zisun Chinese Pharmaceutical Co., Ltd. (Guangzhou, China).

## Preparation of analytical sample of JPHYD for LC-MS/MS analysis

A mixture of 600 g *Salvia miltiorrhiza* Bge. (Danshen), 600 g *Poria cocos* (Schw.) Wolf (Fuling), 400 g *Curcuma phaeocaulis* Valeton (Ezhu), 600 g *Bupleurum chinense* DC. (Chaihu), 400 g *Paeonia suffruticosa* Andr. (Mudanpi), 480 g *Dioscorea oppositifolia* L. (Shanyao), 240 g *Glycyrrhiza uralensis* Fisch. ex DC. (Gancao), 800 g *Panax ginseng* C.A.Mey. (Renshen), and 400 g *Curcuma longa* L. (Yujin), along with 600 g *Atractylodes macrocephala* Koidz. (Baizhu), was prepared. The mixture underwent two rounds of decoction with 51 L water each time for 1 hour. The decoction filtrates were combined and then concentrated to a relative density of 1.08. In a Mini Spray Dryer B-290 (Buchi, Switzerland), spray drying was conducted. The atomization gas flow was adjusted to 3.2 L/min, and the pump speed was set to 3 mL/min. The inlet temperature was 160 °C, and the outlet temperature was 100 °C.

The spray-drying powder of JPHYD (20 mg) was dissolved in a mixture of acetonitrile and 0.1% v/v aqueous formic acid, and subjected to ultrasonic extraction for 30 mins. Subsequently, the solution was passed through a 0.22 μm membrane filter to acquire stock reference solutions of JPHYD, which were subsequently stored at 4 °C.

## Preparation of standard solutions

To prepare the control solutions, weigh the appropriate amounts of *Poricoic* acid A, *Ginsenoside* Rg1, *Tanshinone* ⅡA, and *Saikosaponin* A. Dissolve and dilute the reference metabolites in a 50:50 (v/v) mixture of acetonitrile and 0.1% aqueous formic acid solution. Shake the solutions to ensure uniformity, and adjust the concentrations to 594, 650, 540, and 538 ng/mL, respectively.

## The chromatographic conditions

Analytical sample analysis for standard solutions and stock reference solutions of JPHYD were performed on an ACCELA UHPLC (Thermo Scientific Inc., San Jose, USA) equipped with ACCELA Autosampler and ACCELA 1250 Pump, using a ChromCoreTM 120 C18 column (2.1×50mm, 3μm, Nano Chorm, Shanghai, China). In this study, the mobile phase consisted of a mixture of 0.1% aqueous formic acid (v/v, A) and acetonitrile (B). For elution, a gradient was employed, starting with 60% A from 0 to 7 mins, followed by a decrease to 30% A from 7 to 9 mins, and finally holding at 30% A from 9 to 15 mins. The flow rate was set at 0.4 mL/min, and each injection volume was 5 μL. Throughout the analysis, the column temperature was maintained at a constant 35 °C.

## Mass-spectrometry conditions

TSQ Quantum ACCESS MAX (Accela LC-MS, Thermo Scientific Inc., San Jose, USA) with an electrospray ionization (ESI) source was employed for the qualitative analysis, set to operate in both positive and negative modes. The scanning mode utilized was select reaction monitoring (SRM) mode. The spray voltage was set at 3500 for positive mode and 3000 for negative mode; The capillary temperature was set to 350 °C, and the sheath gas pressure, Collision gas pressure, and Aux gas pressure were adjusted to 40 arb, 1.5m Torr, and 10 arb, respectively.

## Results

Through a thorough literature analysis, we identified four active metabolites with the potential for inhibiting hepatocellular carcinoma metastasis. These metabolites, namely *Poricoic* acid A, *Ginsenoside* Rg1, *Tanshinone* ⅡA, and *Saikosaponin* A, were subjected to qualitative analysis using HPLC-ESI-TSQ-MS. The mass spectrometry parameters for each metabolites are presented in Supplementary Table 1. Additionally, Supplementary Figure 1A displays the ion flow chromatograms of the control samples for the four metabolites, while Supplementary Figure 1B showcases the ion flow chromatograms of JPHYD. The chemical structure of the active metabolites of JPHYD is shown in Supplementary Figure 2.

## References

XIE, C.-F., FENG, K.-L., WANG, J.-N., LUO, R., FANG, C.-K., ZHANG, Y., et al. 2022. Jianpi Huayu decoction inhibits the epithelial-mesenchymal transition of hepatocellular carcinoma cells by suppressing exosomal miR-23a-3p/Smad signaling. Journal of Ethnopharmacology, 294, 115360.https://doi.org/10.1016/j.jep.2022.115360

## 2 The addition of other components by the TCM practitioner

We retrieved the original Chinese herbal prescription data of the patients, and a small subset of patients showed variations in their prescriptions with the presence of the following symptoms: throat dryness, headache, and decreased urine output. To address these symptoms, in addition to administering the JPHYD formula studied in our research, we also added some other Chinese herbal medicines to alleviate the related symptoms (Supplementary Table 2). Importantly, these additional herbal components are unrelated to the main symptoms of postoperative liver cancer patients and do not impact their overall survival rate. This approach ensures the effectiveness of the primary treatment strategy while providing supplementary support for the patients' overall well-being.

**Supplementary Table 1:** Mass spectra properties of four metabolites in JPHYD

| Metabolite name | Q1/Q3 m/z | Tube Lens Voltage | Collision Energy |
| --- | --- | --- | --- |
| *Ginsenoside* Rg_1_ | 823.41→643.50 | 82 | 40 |
| *Saikosaponin* A | 799.69→617.50 | 127 | 41 |
| [*Poricoic* acid A](https://www.chemsrc.com/en/cas/137551-38-3_1198132.html) | 514.36→62.80 | 59 | 80 |
| *Tanshinone* ⅡA | 295.14→277.10 | 72 | 19 |


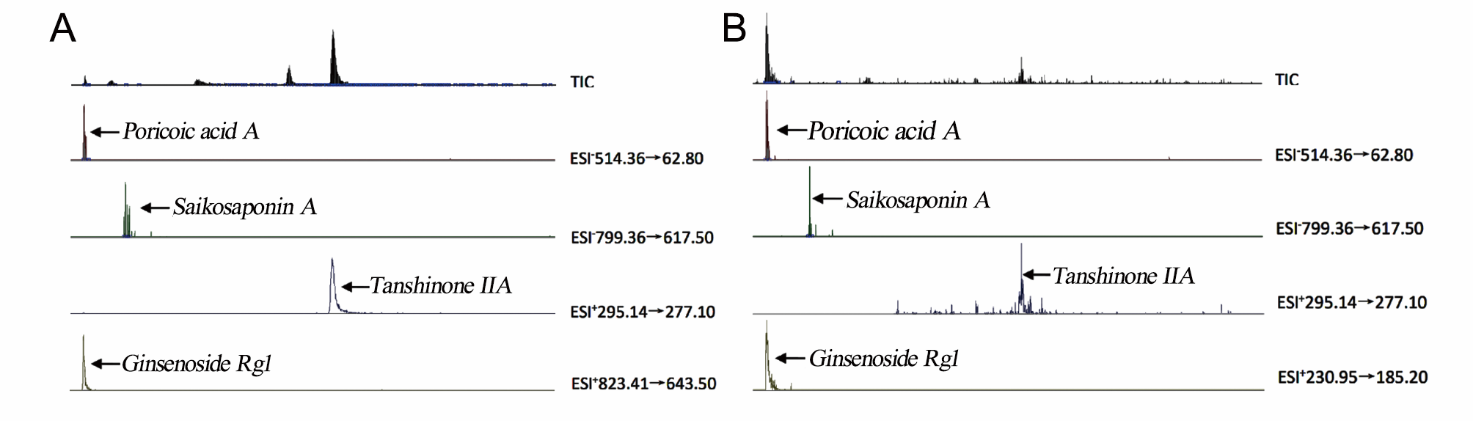


**Supplementary Figure 1:** LC-MS/MS chromatogram of the four reference metabolites(A) and JPHYD(B).


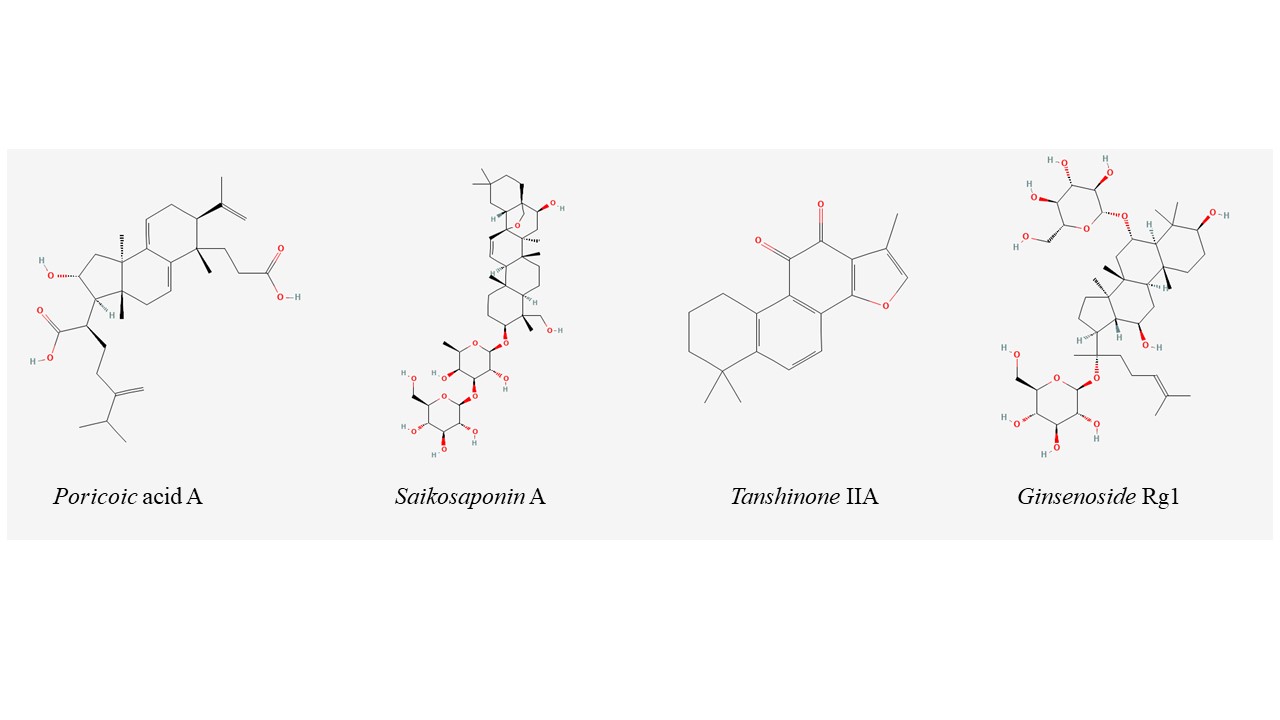


**Supplementary Figure 2:** The chemical structure of active metabolites in JPHYD.

**Supplementary Table 2:** The addition of other components by the TCM practitioner

| **Patient Symptoms** | **Herb name in Chinese** | **Latin name** | **Scientific name** | **Family** | **Parts used** | **Weight （g）** |
| --- | --- | --- | --- | --- | --- | --- |
| Throat itchiness | Juhua | *Chrysanthemi flos* | *Chrysanthemum morifolium* (Ramat.) Hemsl. | Asteraceae | dried capitulum | 10 |
|  | Sangye | *Mori folium* | *Morus alba* L. | Moraceae | dried leaves | 10 |
| Headache | Qianghuo | *Notopterygii rhizoma et radix* | *Notopterygium franchetii* H. de Boiss. | Apiaceae | dried rhizome and roots | 10 |
|  | Tianma | *Gastrodiae rhizoma* | *Gastrodia elata* Blume | Orchidaceae | dried rhizome | 10 |
| Reduced Urination | Jinqiancao | *Desmodii styracifolii herba* | *Desmodium styracifolium* (Osbeck) H.Ohashi & K.Ohashi | Fabaceae | dried aerial part | 10 |
